# Annealing-Regulated Co_3_(PO_4_)_2_ for Enhanced Electrochemical Kinetics in Asymmetric Supercapacitors

**DOI:** 10.3390/molecules31122154

**Published:** 2026-06-18

**Authors:** Pritam J. Morankar, Aviraj M. Teli, Sonali A. Beknalkar

**Affiliations:** 1 School of Chemical Engineering, Yeungnam University, 280 Daehak-ro, Gyeongsan 38541, Republic of Korea; 2 Division of Electronics and Electrical Engineering, Dongguk University-Seoul, Seoul 04620, Republic of Korea

**Keywords:** annealing engineering, electrochemical kinetics, diffusion-controlled charge storage, asymmetric supercapacitor, energy storage

## Abstract

Thermal regulation of electrode materials offers an effective strategy for optimizing electrochemical kinetics in phosphate-based energy-storage systems. In this work, cobalt phosphate (Co_3_(PO_4_)_2_) (CoP) electrodes were directly synthesized on nickel foam through a hydrothermal route and subsequently annealed at different temperatures (300, 400, and 500 °C) to investigate the influence of thermal treatment on structural evolution and supercapacitive behavior. X-ray diffraction confirmed the formation of crystalline CoP, while FESEM analysis revealed a strong dependence of morphology on annealing temperature, with CoP-400 exhibiting a well-developed interconnected plate-like architecture favorable for ion transport. XPS and elemental mapping verified the successful incorporation and uniform distribution of Co, P, and O species. Electrochemical investigations demonstrated that annealing temperature critically governs charge-storage behavior, ion diffusion, and mass transport properties. Among all electrodes, CoP-400 exhibited the best electrochemical performance, delivering a high areal capacitance of 28.62 F/cm^2^ at 20 mA/cm^2^, together with the highest ionic diffusion coefficient, lowest equivalent series resistance (0.39 Ω), and dominant diffusion-controlled charge-storage contribution (89%). Furthermore, CoP-400 retained 84.44% capacitance after 12,000 cycles. An asymmetric supercapacitor assembled using CoP-400//AC achieved an areal capacitance of 302 mF/cm^2^, an energy density (ED) of 0.094 mWh/cm^2^, and excellent cycling stability. These findings highlight annealing-engineered CoP as a promising electrode material for high-performance asymmetric supercapacitors.

## 1. Introduction

The way electrical energy is generated, distributed, and consumed is undergoing a profound transformation. The increasing penetration of renewable power sources, decentralized energy networks, and electrified transportation has shifted attention from energy generation alone to efficient energy management. Unlike conventional power systems, renewable sources such as solar and wind operate under inherently variable conditions, often creating a mismatch between energy supply and demand [1,2]. This has intensified the need for electrochemical energy-storage technologies that can rapidly capture and deliver energy while maintaining stable long-term operation under repeated cycling [3]. Among the available electrochemical energy-storage technologies, supercapacitors have attracted considerable attention because of their high-power density, rapid charge–discharge capability, and excellent cycling durability. These characteristics make them particularly attractive for applications requiring fast energy delivery, including portable electronics, regenerative braking systems, pulse-power devices, and backup power modules. However, despite these advantages, the relatively low ED of supercapacitors remains a major challenge that limits their broader practical deployment [4,5]. Since the electrochemical performance of supercapacitors is largely governed by the nature of the electrode material, the development of advanced electrode materials remains central to improving device performance [6,7].

Carbon-based materials such as activated carbon, graphene, and carbon nanotubes store charge mainly through electric double-layer capacitance and generally exhibit excellent electrical conductivity and structural stability [8]. However, their energy-storage capability remains limited because charge accumulation is largely confined to surface adsorption. In contrast, transition-metal-based Faradaic materials store charge through rapid and reversible redox reactions involving multiple oxidation states, offering considerably higher charge-storage capability [9]. Transition-metal oxides, hydroxides, sulfides, phosphides, and related compounds have therefore been widely explored as promising electrode materials. Nevertheless, their practical performance is often limited by sluggish interfacial charge transfer, incomplete electrolyte penetration, and gradual structural degradation during prolonged cycling [10]. Among the emerging pseudocapacitive materials, transition-metal phosphates have attracted increasing attention because they combine electrochemical activity with relatively robust structural frameworks [11]. The phosphate groups modify the local electronic environment of transition-metal centers and help preserve structural stability during repeated redox cycling. Several phosphate-based materials, including nickel phosphate (Ni_3_(PO_4_)_2_), manganese phosphate (Mn_3_(PO_4_)_2_), iron phosphate (FePO_4_), and nickel–cobalt phosphate (NiCoPO_4_), have shown encouraging electrochemical behavior in energy-storage applications. Within this family, CoP has emerged as a promising candidate because cobalt provides electrochemically active redox centers, while the phosphate framework contributes to chemical robustness under alkaline conditions [12,13].

Recent studies have highlighted the potential of CoP-based materials for supercapacitor applications. Xiao et al. synthesized CoP nanorods through a hydrothermal route and reported a specific capacitance of 1826.7 F/g at 1 A/g with 86.8% capacitance retention after 10,000 cycles. The assembled COP//rGO asymmetric device delivered an ED of 37.0 Wh/kg and retained 92.3% of its initial capacitance after 5000 cycles [14]. Shinde et al. prepared porous one-dimensional Co_2_P_2_O_7_ nanobelts and observed that the sample synthesized at 150 °C exhibited the best electrochemical performance, delivering 1766 F/g at 5 mV/s. Their Co_2_P_2_O_7_//activated carbon asymmetric device further delivered an ED of 83.16 Wh/kg at a power density (PD) of 9.35 kW/kg [15]. Sankar et al. reported one-dimensional Co_3_(PO_4_)_2_ nanograss structures grown directly on nickel foam, where the assembled hybrid supercapacitor achieved an ED of 26.66 Wh/kg at 750 W/kg with 80% capacitance retention after 6000 cycles [16]. Similarly, Ganth et al. prepared CoP thin films by chemical bath deposition, and the optimized sample exhibited a specific capacitance of 678 F/g at 10 mV/s together with 90.46% capacitance retention after 10,000 cycles [17]. These reports clearly demonstrate the promise of CoP for electrochemical energy storage. At the same time, the broad variation in reported electrochemical performance suggests that the charge-storage behavior of CoP is highly sensitive to its structural state. Crystallinity, defect population, interparticle connectivity, and electrolyte-accessible active sites collectively determine how effectively the intrinsic redox centers participate in electrochemical reactions [18]. Among the various parameters that influence these structural characteristics, thermal treatment is particularly important. In nanostructured phosphate systems, annealing governs crystallographic development, defect redistribution, grain-boundary continuity, and surface reconstruction [19]. Insufficient thermal treatment may preserve a highly disordered framework with poor electronic connectivity, whereas excessive annealing can induce particle coalescence and structural densification, thereby reducing electrochemically accessible surface area and extending ion-diffusion pathways. Therefore, the electrochemical response of CoP depends on achieving an appropriate balance between structural ordering and kinetically accessible transport channels [20].

Despite the growing interest in CoP-based electrodes, most previous studies have focused primarily on synthesis routes, compositional tuning, and morphology engineering. Comparatively less attention has been devoted to understanding how post-synthetic thermal treatment governs electrochemical kinetics. In particular, the relationship between annealing-induced structural evolution and the relative contributions of surface-controlled and diffusion-governed charge-storage processes remains insufficiently understood. Establishing this correlation is important for developing a clearer structure–property relationship and for rationally optimizing electrochemical performance. Motivated by these considerations, CoP nanostructures were subjected in the present work to controlled post-synthetic annealing in order to investigate the influence of thermal treatment on structural evolution and electrochemical behavior. The effect of annealing temperature on crystallographic characteristics, surface morphology, and electrochemically accessible architecture was systematically examined. Particular attention was directed toward understanding the electrochemical charge-storage behavior of thermal reconstruction through diffusion analysis, charge-transfer behavior, b-value evaluation, and separation of capacitive and diffusion-controlled contributions. The thermally optimized CoP-400 electrode was subsequently employed as the positive electrode in an asymmetric supercapacitor to evaluate practical device-level performance in terms of rate capability, energy–power characteristics, and cycling durability.

## 2. Experiments

### 2.1. Materials

Cobalt nitrate hexahydrate (Co(NO_3_)_2_·6H_2_O), ammonium dihydrogen phosphate (NH_4_H_2_PO_4_), urea (CO(NH_2_)_2_), hydrochloric acid (HCl), ethanol, and deionized (DI) water were used as received without further purification. Nickel foam (NF, thickness ~1.6 mm) was used as the conductive substrate for electrode preparation.

### 2.2. Hydrothermal Synthesis of CoP Electrodes

**Figure 1:**
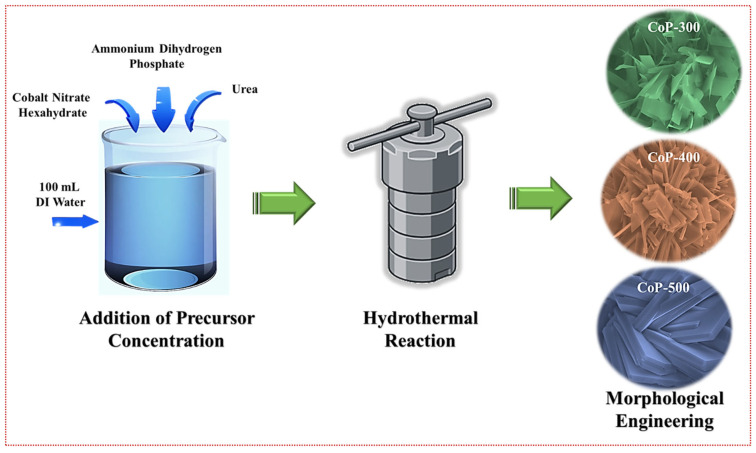
Schematic illustration of the hydrothermal synthesis and annealing process for CoP-300, CoP-400, and CoP-500 electrodes.

CoP electrodes were directly grown on nickel foam (NF) through a hydrothermal synthesis process. In a typical preparation, 1.455 g of Co(NO_3_)_2_·6H_2_O (5 mmol), 0.575 g of NH_4_H_2_PO_4_ (5 mmol), and 0.600 g of CO(NH_2_)_2_ (10 mmol) were dissolved in 100 mL of deionized (DI) water under continuous magnetic stirring. The precursor solution was stirred for 30 min until a clear and homogeneous solution was obtained. NF substrates (1 × 2 cm^2^) were cleaned sequentially by ultrasonication in dilute HCl, DI water, and ethanol to remove surface oxides and impurities. The cleaned NF was then vertically immersed into the prepared precursor solution and transferred into a Teflon-lined stainless-steel autoclave. The hydrothermal reaction was conducted at 170 °C for 12 h, followed by natural cooling to room temperature. After completion of the reaction, the CoP-coated NF electrodes were collected, thoroughly washed several times with DI water and ethanol to remove loosely attached residues, and dried at 80 °C overnight. The obtained hydrothermal electrodes were subsequently annealed in air at different temperatures of 300, 400, and 500 °C for 2 h with a heating rate of 2 °C min^−1^, followed by natural cooling to room temperature. The annealed samples were designated as CoP-300, CoP-400, and CoP-500, respectively. A schematic illustration of the hydrothermal growth of CoP on nickel foam followed by post-annealing treatment is depicted in Figure 1.

### 2.3. Sample Characterization and Electrochemical Measurements

The phase structure and crystallographic characteristics of the prepared COP electrodes were examined by X-ray diffraction (XRD: PANalytical, Almelo, The Netherlands) using Cu Kα radiation (λ = 1.5406 Å). Surface morphology and annealing-induced microstructural evolution were investigated by field-emission scanning electron microscopy (FESEM, Hitachi S-4800, Chiyoda, Tokyo, Japan). The elemental composition and spatial distribution of the constituent elements were analyzed using energy-dispersive X-ray spectroscopy (EDS) coupled with the FESEM system. The surface chemical environment and oxidation states of the elements were further examined by X-ray photoelectron spectroscopy (XPS: UK analysis system, Cheshire, UK). All XPS spectra were calibrated using the internal charge compensation and instrumental calibration procedure. Electrochemical measurements were carried out using a BioLogic electrochemical workstation (BioLogic Science Instruments, Seyssinet-Pariset, France) in a conventional three-electrode configuration with 2 M KOH aqueous electrolyte. In this configuration, nickel foam primarily served as a conductive current collector, while the electrochemical response was predominantly governed by the directly grown CoP active layer. Cyclic voltammetry (CV), galvanostatic charge–discharge (GCD), and electrochemical impedance spectroscopy (EIS) measurements were performed to evaluate the charge-storage characteristics, reaction kinetics, and cycling stability of the prepared electrodes.

## 3. Results and Discussion

### 3.1. XRD Analysis

**Figure 2:**
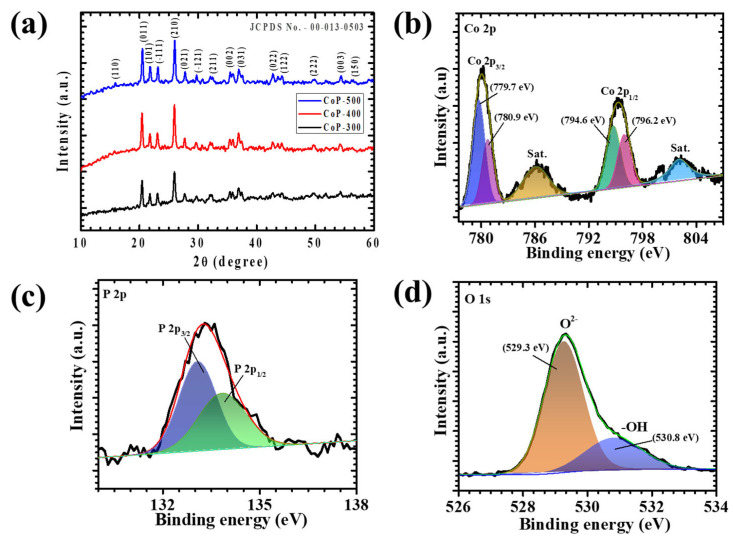
(**a**) XRD patterns of CoP-300, CoP-400, and CoP-500; high-resolution XPS spectra of optimized CoP-400 showing (**b**) Co 2p, (**c**) P 2p, and (**d**) O 1s spectra.

The crystal structure and phase evolution of the thermally treated CoP electrodes were examined using XRD, and the corresponding patterns of CoP-300, CoP-400, and CoP-500 are shown in Figure 2a. All diffraction peaks are well indexed to crystalline CoP, and match well with the standard JCPDS card No. 00-013-0503, confirming the successful formation of the CoP phase after hydrothermal growth and subsequent thermal treatment. The diffraction peaks appearing at approximately 15.81°, 20.8°, 21.82°, 23.15°, 25.87°, 27.78°, 29.65°, 31.93°, 32.40°, 35.43°, 35.98°, 36.93°, 42.66°, 44.21°, 49.81°, 54.81°, and 56.34° are assigned to the (110), (011), (101), (−111), (210), (021), (−121), (220), (211), (002), (−221), (031), (022), (122), (222), (003) and (150) planes, respectively, which are characteristic of CoP. The absence of additional diffraction peaks indicates good phase purity without detectable secondary phases. The annealing temperature strongly affects the crystallographic characteristics of the electrodes. The CoP-300 sample exhibits relatively broad and less intense diffraction peaks, indicating lower crystallinity and smaller crystallite domains. Upon increasing the annealing temperature to 400 °C (CoP-400), the diffraction peaks become sharper and more intense, suggesting improved crystal growth and enhanced structural ordering. For the CoP-500 electrode, the peaks become more pronounced and better defined, reflecting enhanced crystallinity and improved long-range ordering of the CoP structure. The gradual sharpening and increased intensity of the diffraction peaks with increasing annealing temperature suggest thermally induced crystallite growth and structural stabilization, which may influence ion diffusion and electrochemical charge-transfer behavior [21].

### 3.2. XPS Analysis

The surface elemental composition and chemical states of the optimized CoP-400 electrode were further examined by XPS, and the corresponding high-resolution spectra are shown in Figure 2b–d. The deconvoluted spectra confirm the presence of Co, P, and O elements, validating the successful formation of CoP after thermal treatment. The high-resolution Co 2p spectrum in Figure 2b exhibits two major spin–orbit doublets corresponding to Co 2p_3/2_ and Co 2p_1/2_. The deconvoluted peaks centered at approximately 779.7 and 780.9 eV are assigned to the Co 2p_3/2_ region, while the peaks located at around 794.6 and 796.2 eV correspond to Co 2p_1/2_, indicating the coexistence of cobalt species in oxidized states. In addition, the appearance of shake-up satellite peaks at approximately 786.0 and 803.0 eV further confirms the presence of Co^2+^ species, which is characteristic of CoP compounds [22]. The observed peak positions and satellite features are consistent with cobalt coordinated within a phosphate framework. The P 2p spectrum shown in Figure 2c can be deconvoluted into two characteristic peaks centered at approximately 133.4 eV and 134.2 eV, corresponding to P 2p_3/2_ and P 2p_1/2_, respectively. These binding energies are characteristic of phosphate (PO_4_^3−^) groups, confirming the successful incorporation of phosphorus into the CoP structure. The absence of peaks at lower binding energies indicates that no metallic phosphide phase was formed. As displayed in Figure 2d, the high-resolution O 1s spectrum consists of two fitted components located at approximately 529.3 eV and 530.8 eV. The lower binding energy peak is attributed to lattice oxygen (Co–O/P–O bonding) associated with the CoP framework, whereas the higher binding energy component corresponds to surface hydroxyl groups or adsorbed oxygen species. The presence of these oxygen-containing functional species may facilitate electrolyte penetration and improve interfacial electrochemical reactions. The XPS results strongly support the formation of CoP with well-defined Co, P, and O chemical environments in the optimized CoP-400 electrode, in agreement with the phase analysis obtained from XRD [23].

### 3.3. FESEM and Elemental Mapping Analysis

**Figure 3:**
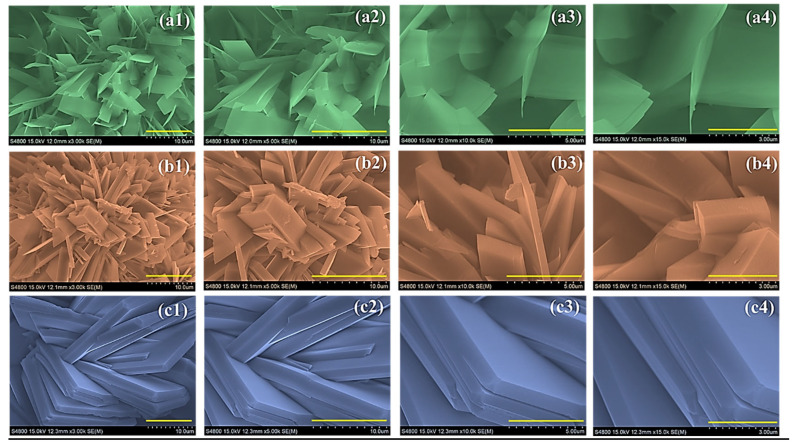
FESEM images of (**a1**–**a4**) CoP-300, (**b1**–**b4**) CoP-400, and (**c1**–**c4**) CoP-500 at different magnifications.

The surface morphology of the thermally treated CoP electrodes was analyzed using FESEM, and the obtained images are presented in Figure 3. The micrographs correspond to CoP-300 (Figure 3(a1–a4)), CoP-400 (Figure 3(b1–b4)), and CoP-500 (Figure 3(c1–c4)), revealing the effect of annealing temperature on the structural growth and surface features of the prepared electrodes. The CoP-300 electrode (Figure 3(a1–a4)) shows a loosely arranged morphology consisting of randomly distributed plate-like nanosheets. The structure appears relatively less organized, with thin sheets stacked irregularly and several empty spaces between adjacent layers. At higher magnification, the nanosheets appear less compact and not fully developed, suggesting limited crystal growth at the lower annealing temperature. For CoP-400 sample (Figure 3(b1–b4)), the morphology changes significantly, showing a more uniform and well-grown structure made up of interconnected plate-like units arranged in a flower-like architecture. The nanosheets appear thicker, smoother, and more clearly defined than those of CoP-300. In the high-magnification images, sufficient spacing between neighboring plates can still be observed, which may allow easier electrolyte access and faster ion movement during electrochemical reactions. The structure appears both compact and open at the same time, which is beneficial for maintaining active surface area while improving electron transport. In contrast, the CoP-500 sample (Figure 3(c1–c4)) exhibits noticeably larger and thicker plate-like structures. With increasing annealing temperature, the sheets become more densely stacked and highly compact. The individual plates are well developed, but excessive growth leads to stronger aggregation and reduced open spaces within the structure. This may decrease electrolyte accessibility and limit the number of exposed active sites. The FESEM results suggest that annealing temperature strongly affects the morphology of CoP. Compared with CoP-300 and CoP-500, the CoP-400 electrode shows a more favorable structure with interconnected nanosheets, good surface exposure, and sufficient open channels, which can support efficient ion transport and electrochemical activity.

**Figure 4:**
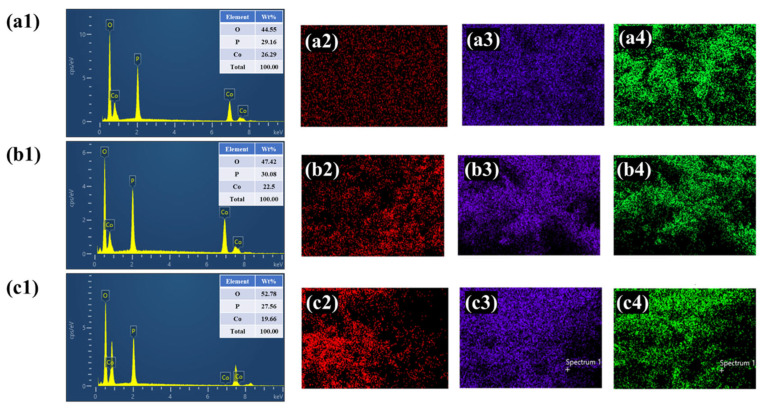
EDS spectra and elemental mapping images of (**a1**–**a4**) CoP-300, (**b1**–**b4**) CoP-400, and (**c1**–**c4**) CoP-500.

The elemental composition of the prepared CoP-300, CoP-400, and CoP-500 electrodes was analyzed using EDS coupled with the FESEM instrument. The EDS spectra presented in Figure 4(a1–c1) exhibit distinct peaks corresponding to Co, P, and O, confirming the successful formation of the CoP structure without the presence of detectable impurity elements. The observed elemental signals verify the successful incorporation of cobalt, phosphorus, and oxygen within the prepared electrodes. To further investigate the elemental distribution, EDS elemental mapping was carried out for all prepared samples. As shown in Figure 4(a2–a4,b2–b4,c2–c4), the mapping images demonstrate a uniform distribution of O, P, and Co across the surface of CoP-300, CoP-400, and CoP-500, respectively. No obvious elemental segregation or agglomeration is observed, indicating homogeneous elemental incorporation throughout the electrode surface. The well-dispersed elemental arrangement suggests that the synthesis process promotes uniform nucleation and growth of the CoP structure. In particular, the optimized CoP-400 sample exhibits highly uniform elemental distribution, which may contribute to improved structural stability and efficient electrochemical charge transfer.

## 4. Electrochemical Analysis

**Figure 5:**
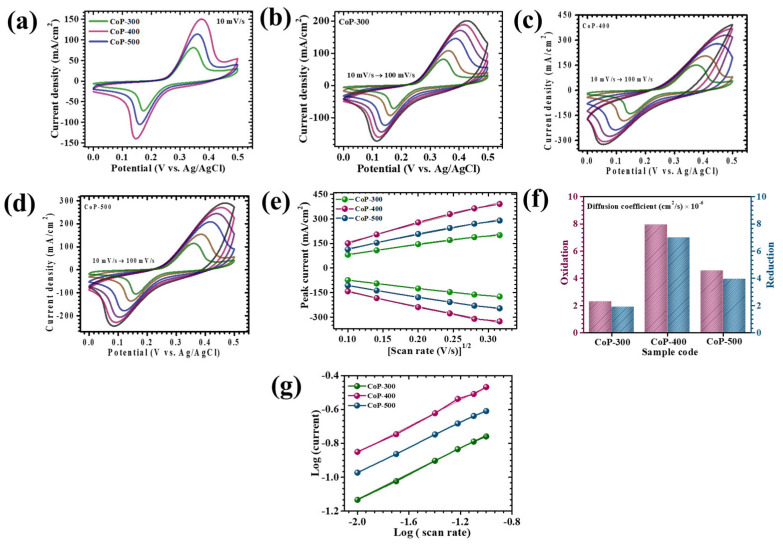
CV and kinetic analysis of CoP electrodes: (**a**) CV curves at 10 mV/s, (**b**–**d**) CV profiles at different scan rates, (**e**) peak current versus square root of scan rate, (**f**) diffusion coefficients, (**g**) b-value analysis.

Figure 5a illustrates the CV characteristics of the annealed CoP electrodes measured between 0.0 and 0.5 V (vs. Ag/AgCl) at a scan rate of 10 mV/s. The CV curves are dominated by a pair of well-resolved oxidation (~0.36 V) and reduction (~0.15 V) peaks, indicating that the energy-storage process proceeds primarily through reversible Faradaic reactions rather than ideal double-layer capacitance. These redox transitions are associated with reversible OH^−^-assisted oxidation-state changes of cobalt species under alkaline conditions. Considering the presence of a single redox peak pair, the electrochemical response may arise from overlapping cobalt redox processes rather than distinct individual oxidation reactions. The appearance of distinct redox features with appreciable current density confirms the electroactive nature of the phosphate framework and its ability to sustain rapid electron-transfer processes during repeated cycling [24]. A substantial variation in electrochemical response is observed with annealing temperature, demonstrating the critical role of thermal treatment in governing the structural and kinetic characteristics of the electrodes. Among all samples, CoP-400 exhibits the highest current response and the broadest enclosed CV profile, reflecting superior charge-storage capability and enhanced electrochemical activity. The improved behavior can be attributed to the formation of a structurally optimized CoP network possessing sufficient crystallographic ordering together with accessible ion-transport channels. Such a configuration facilitates rapid electrolyte infiltration and enables efficient utilization of redox-active cobalt centers. Moreover, the relatively low polarization of CoP-400 suggests accelerated reaction kinetics and improved reversibility of the Faradaic process [25].

The influence of scan rate on electrochemical behavior was further examined over the range of 10–100 mV/s as displayed in Figure 5b–d. Increasing the scan rate leads to a proportional enhancement in anodic and cathodic current densities while preserving the fundamental redox characteristics, indicating robust electrochemical stability and favorable ion-transport dynamics. Although slight peak displacement is observed at higher sweep rates due to internal resistance and kinetic limitations, the overall retention of peak shape demonstrates rapid charge propagation within the electrode matrix. The persistence of these Faradaic features even under fast polarization conditions confirms efficient electron mobility and electrolyte accessibility throughout the active material. Notably, the electrochemical response of CoP-400 remains considerably more stable and intense than that of CoP-300 and CoP-500 across the entire scan-rate range. This behavior suggests that annealing at 400 °C generates an optimal physicochemical environment for charge storage by balancing crystallinity and structural openness. The interconnected porous arrangement formed at this temperature likely provides shortened diffusion pathways for electrolyte ions while simultaneously maintaining adequate electrical connectivity between active domains. As a result, both ion diffusion and electron transport occur more effectively, leading to enhanced pseudocapacitive kinetics [26]. In contrast, the electrode annealed at 300 °C suffers from insufficient structural evolution, which may result in incomplete phosphate phase development and weak interparticle connectivity. These characteristics restrict efficient charge transport and limit the participation of electroactive sites during redox reactions. On the other hand, excessive thermal exposure at 500 °C promotes particle fusion and structural compactness, diminishing surface accessibility and reducing the number of ion-active interfaces. The resulting decline in electrolyte penetration and diffusion efficiency adversely affects the overall capacitive behavior [27]. The comparative electrochemical analysis therefore demonstrates that the superior performance of CoP-400 originates from a favorable synergy between structural organization, ion-accessible porosity, and reversible cobalt redox chemistry.

The charge-storage behavior and ion-transport characteristics of the annealed CoP electrodes was further analyzed through the relationship between peak current and scan rate. As presented in Figure 5e, both anodic and cathodic peak currents exhibit an excellent linear dependence with the square root of scan rate (*ν*^1/2^) for all CoP electrodes, indicating that the charge-storage mechanism is predominantly governed by diffusion-assisted Faradaic reactions. Under alkaline conditions, the electrochemical response is associated with reversible OH^−^-assisted cobalt redox transitions contributing to the overall charge-storage behavior [24]. Consequently, the efficiency of ion migration through the porous electrode framework critically governs the reaction rate and charge-storage capability. For quantitative evaluation of ionic transport behavior, the apparent diffusion coefficients (D) of the electroactive species were estimated using the Randles–Sevcik equation [28] (1):

(1) $$D^{1/2}=\frac{i_{p}}{2.69\times 10^{5}\times n^{3/2}\times A\times C\times v^{1/2}}$$

where *i_p_* represents the peak current extracted from the CV curves, *n* is the number of electrons participating in the redox reaction, *A* corresponds to the effective electrochemical surface area, *C* denotes the concentration of active ionic species in the electrolyte, and *ν* refers to the scan rate. The diffusion coefficients were calculated at a scan rate of 10 mV/s to maintain identical electrochemical conditions for all investigated electrodes. The obtained values are summarized in Table 1 and comparatively illustrated in Figure 5f. Among the investigated samples, CoP-400 exhibits the highest diffusion coefficient, signifying remarkably improved ionic mobility and accelerated charge-transfer kinetics. The superior diffusion behavior of CoP-400 can be attributed to its optimized thermally evolved architecture, which simultaneously promotes interconnected ion-transport pathways and efficient electrical conduction. Such an optimized configuration substantially reduces diffusion resistance and enhances the utilization of redox-active sites during the electrochemical process.

To obtain deeper insight into the intrinsic charge-storage mechanism of the thermally engineered CoP electrodes, the electrochemical charge-storage behavior was systematically evaluated using power-law analysis derived from CV data collected at different scan rates. The relationship between peak current (*i*) and scan rate (*v*) follows the classical power-law expression (2) [29]:

(2) $$i\;={av}^{b}$$

where *a* and *b* are adjustable parameters and the exponent *b* serves as a key indicator of the dominant charge-storage mechanism. In general, *b* ≈ 0.5 corresponds to diffusion-regulated Faradaic behavior, whereas *b* ≈ 1 indicates a surface-controlled capacitive process [30]. The *b*-values for the CoP electrodes were extracted from the slopes of the linear fitting plots of log(*i*) versus log(*v*) (Figure 5g). The obtained values for CoP-300, CoP-400, and CoP-500 were found to remain considerably closer to 0.5 (Table 1), confirming that the electrochemical response of all electrodes is primarily governed by diffusion-assisted pseudocapacitive reactions involving electrolyte-ion transport within the active framework. Notably, CoP-400 exhibits a comparatively closer to 0.5 *b*-value than CoP-300 and CoP-500, indicating a more favorable balance between diffusion-mediated redox activity and surface-controlled charge storage. This behavior suggests that annealing at 400 °C effectively optimizes the electrochemical accessibility of cobalt active sites while simultaneously preserving rapid ionic diffusion throughout the porous electrode network.

**Figure 6:**
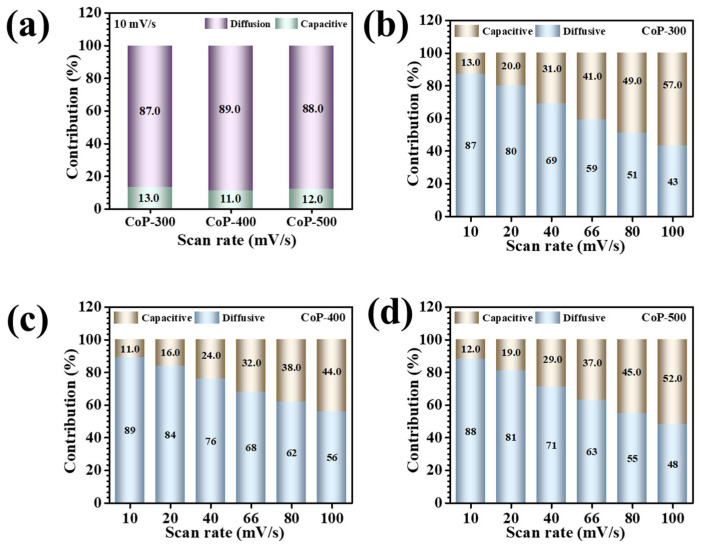
Charge-storage kinetics of CoP electrodes: (**a**) capacitive and diffusion-controlled contributions at 10 mV/s, and (**b**–**d**) contribution analysis at different scan rates.

To further distinguish the relative contributions from capacitive and diffusion-controlled processes, the total current response was quantitatively separated according to the following relationship (3) [31]:

(3) $$i\left(V\right)=k_{1}v{\;+\;k_{2}v}^{1/2}$$

where the *k*_1_*v* component corresponds to surface-controlled capacitive charge storage, while the *k*_2_*v*^1/2^ term represents diffusion-governed Faradaic reactions. By fitting the electrochemical response at various potentials, the individual contributions from capacitive and diffusion-controlled processes were quantitatively estimated for each electrode. The total stored charge can therefore be expressed as (4) [31]:

(4) $$Q_{t}=Q_{s}+Q_{d}$$

where *Q_s_* represents the capacitive contribution and *Q_d_* denotes the diffusion-assisted charge-storage component. The kinetic analysis reveals that diffusion-controlled processes dominate the overall charge-storage behavior for all CoP electrodes at lower scan rates (10 mV/s; Figure 6a), confirming that ion insertion/extraction within the electroactive framework plays a major role in the electrochemical response. The CoP-400 electrode exhibits the highest diffusion-governed contribution of 89%, demonstrating substantially improved ion-transport capability and enhanced electrochemical utilization of the active material. Furthermore, increasing the scan rate gradually enhances the relative capacitive contribution for all electrodes (Figure 6b–d). This trend originates from kinetic limitations imposed under rapid polarization conditions, where electrolyte ions cannot sufficiently penetrate the deeper electroactive regions within the limited time scale of the measurement [32]. Consequently, charge storage becomes increasingly dominated by surface-controlled processes at higher sweep rates. Nevertheless, CoP-400 maintains a substantially larger diffusion-controlled fraction even at elevated scan rates, highlighting its superior structural capability to alleviate ion-transport limitations and sustain efficient Faradaic kinetics under fast charge–discharge operation. In contrast, CoP-300 exhibits comparatively weaker diffusion contribution due to insufficient crystallization and incomplete development of conductive ion-transport pathways at lower annealing temperature. Conversely, excessive thermal treatment in CoP-500 induces structural densification and particle agglomeration, which restrict electrolyte infiltration and reduce electrochemically accessible surface area. These structural limitations hinder effective ion migration and suppress the diffusion-assisted pseudocapacitive response. Overall, the kinetic investigation clearly demonstrates that annealing-temperature modulation critically governs the charge-storage dynamics of CoP electrodes. In particular, CoP-400 achieves an optimal structural configuration that maximizes electrolyte accessibility, and promotes highly reversible diffusion-controlled pseudocapacitance, thereby resulting in superior electrochemical kinetics and enhanced supercapacitive performance [33].

**Figure 7:**
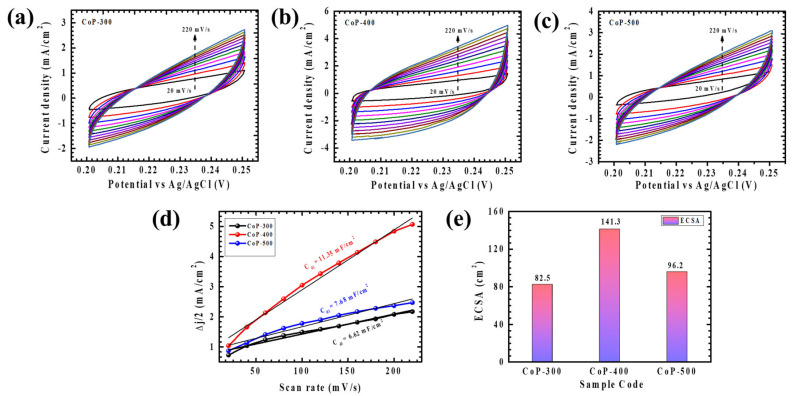
(**a**–**c**) CV curves of CoP-300, CoP-400, and CoP-500 electrodes at different scan rates in the non-faradaic region, (**d**) linear fitting for Cdl determination, and (**e**) calculated ECSA values.

The electrochemically active surface area (ECSA) of the CoP-300, CoP-400, and CoP-500 electrodes was estimated from CV measurements conducted at various scan rates within a non-faradaic potential region (Figure 7a–c). Selecting this potential range minimizes faradaic contributions and allows the current response to originate predominantly from electric double-layer charging. The double-layer capacitance (*C_dl_*) values were extracted from the capacitive current response and used to determine the ECSA according to Equation (5) (Figure 7d):

(5) $$ECSA=\frac{c_{dl}}{C_{s}}$$

where *C_dl_* is the measured double-layer capacitance and *C_s_* is the specific capacitance of a flat surface in 1 M KOH (0.04 mF^−2^). The calculated ECSA values for CoP-300, CoP-400, and CoP-500 were 82.5, 141.3, and 96.2 cm^2^, respectively (Figure 7e). The highest ECSA observed for CoP-400 indicates greater electrochemical accessibility, which can be associated with its interconnected plate-like units arranged in a flower-like architecture. Such a structure promotes enhanced electrolyte contact and provides more exposed active sites, contributing to improved electrochemical behavior.

**Figure 8:**
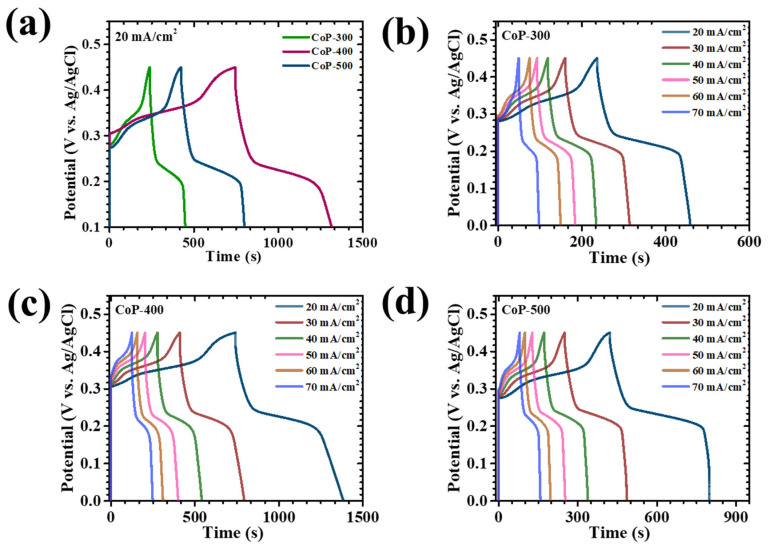
GCD analysis of CoP electrodes: (**a**) GCD curves at 20 mA/cm^2^, and (**b**–**d**) GCD profiles at different current densities.

The GCD behavior of the annealed CoP electrodes was comprehensively investigated to evaluate their charge-storage capability, reaction reversibility, and high-rate electrochemical characteristics. Figure 8a compares the GCD profiles of CoP-300, CoP-400, and CoP-500 recorded at a current density of 20 mA/cm^2^ within the operating potential window (0.0 to 0.45 V). Distinct differences in discharge duration and voltage response are clearly observed among the electrodes, indicating that annealing temperature strongly influences the electrochemical kinetics and utilization efficiency of the active material. To further probe the current-dependent behavior, GCD measurements were subsequently performed over a broad current density range for all samples (20–70 mA/cm^2^), as shown in Figure 8b–d. The obtained charge–discharge curves display obvious deviations from ideal linear triangular behavior and instead exhibit characteristic nonlinear voltage profiles accompanied by discernible discharge plateaus. Such electrochemical characteristics confirm that the energy-storage process in CoP electrodes is predominantly governed by diffusion-assisted Faradaic reactions involving reversible cobalt redox transitions in alkaline electrolyte. The nonlinearity of the discharge curves arises from the continuous oxidation and reduction processes associated with Co^2+^/Co^3+^ and Co^3+^/Co^4+^ conversion reactions, demonstrating the battery-type pseudocapacitive nature of the material [34]. Among all investigated electrodes, CoP-400 exhibits the longest discharge duration at identical current densities, directly reflecting its superior charge-storage capability and enhanced electrochemical accessibility of active cobalt sites. The gradual voltage decay and highly symmetric charge–discharge characteristics further suggest efficient reaction reversibility with minimal kinetic polarization during repeated cycling. The enhanced electrochemical response of CoP-400 can be attributed to the favorable structural evolution induced by annealing at 400 °C, where an optimal balance between crystallinity and porous architecture is achieved. Such a thermally optimized framework promotes rapid electrolyte permeation while simultaneously facilitating efficient electron transport throughout the electrode matrix.

**Figure 9:**
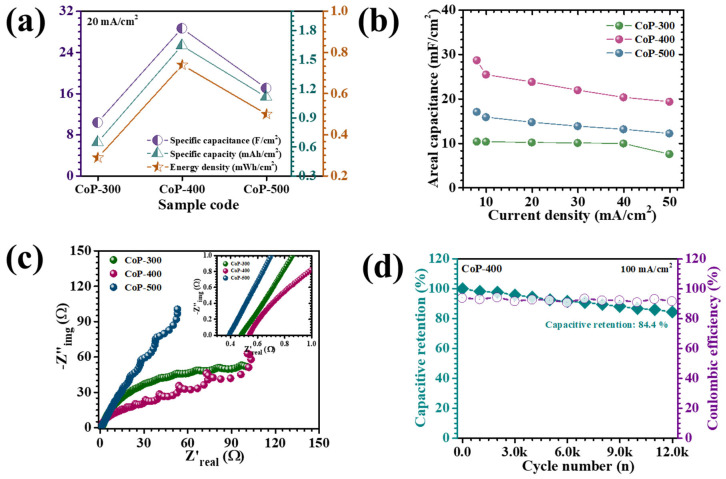
Electrochemical performance of CoP electrodes: (**a**) areal capacitance, (**b**) energy-storage parameters, (**c**) Nyquist plots, and (**d**) cycling stability of CoP-400.

To quantitatively assess the electrochemical storage properties, the areal capacitance (*C_A_*), ED, and PD were calculated using equations adapted for nonlinear pseudocapacitive discharge behavior (6)–(8) [35,36]:

(6) $$C_{A}=\frac{I\times 2\times \int V\left(t\right)dt}{A\times {\left(dV\right)}^{2}}$$

(7) $$ED=\frac{1}{2\times 3600}\;C_{A}\times {dV}^{2}$$

(8) $$PD=\frac{ED\times 3600}{dt}$$

where *I* represents the discharge current, *∫V(t)dt* corresponds to the integrated discharge area accounting for the nonlinear discharge characteristics, *A* denotes the electrochemically active area, *dV* is the operating potential window, and *dt* represents the discharge time. This analytical approach is particularly important for CoP electrodes because conventional linear capacitance calculations cannot accurately describe the nonlinear voltage behavior associated with diffusion-controlled Faradaic charge storage. At current density of 20 mA/cm^2^, CoP-300, CoP-400, and CoP-500 delivered areal capacitances of 10.31, 28.62, and 16.98 F/cm^2^, respectively (Table 2 and Figure 9a). The calculated electrochemical parameters reveal that CoP-400 delivers the highest areal capacitance among all electrodes, confirming the beneficial role of optimized annealing temperature in maximizing electrochemical activity. The superior capacitance of CoP-400 originates from the synergistic contribution of multiple structural and kinetic advantages. First, the thermally optimized architecture exposes a larger number of electrochemically accessible cobalt active sites capable of participating in reversible redox reactions. Second, improved crystallographic ordering enhances electronic conductivity and minimizes charge-transfer resistance across the electrode framework. Third, the interconnected porous morphology facilitates rapid OH^−^ diffusion by providing shortened ion-transport pathways and improved electrolyte infiltration into deeper electroactive regions [37,38]. As expected, all electrodes exhibit a gradual decrease in capacitance with increasing current density due to insufficient time for complete ion diffusion into the inner electroactive regions under rapid charge–discharge conditions [39]. Nevertheless, CoP-400 maintains excellent capacitance retention even at high current densities, highlighting its robust structural stability and efficient reaction kinetics (Figure 9b). The superior rate capability demonstrates that the optimized CoP framework effectively mitigates diffusion limitations and preserves rapid charge-transfer processes under aggressive operating conditions.

EIS was employed to evaluate the charge-transfer resistance and ion-transport behavior of the annealed CoP electrodes. The Nyquist plots (Figure 9c) exhibit a semicircle in the high-frequency region followed by an inclined line at lower frequencies, corresponding to interfacial charge-transfer processes and electrolyte-ion diffusion, respectively. The high-frequency intercept on the real axis represents the equivalent series resistance (ESR), which includes contributions from electrolyte resistance, intrinsic electrode resistance, and electrode/current collector contact resistance [40]. The calculated ESR values for CoP-300, CoP-400, and CoP-500 were found to be 0.53 Ω, 0.39 Ω, and 0.47 Ω, respectively (Table 1). Among the investigated electrodes, CoP-400 exhibits the lowest ESR value along with the smallest semicircle, indicating reduced internal resistance and faster Faradaic charge-transfer kinetics. Furthermore, the steeper low-frequency slope of CoP-400 suggests improved OH^−^ ion diffusion and enhanced electrolyte accessibility within the electrode framework. The superior impedance characteristics of CoP-400 are attributed to its optimized porous architecture and balanced crystallinity achieved at 400 °C, which collectively facilitate efficient electron transport and rapid ion migration. In contrast, CoP-300 suffers from poor crystallization, whereas CoP-500 undergoes structural densification and particle agglomeration, resulting in comparatively higher resistance and sluggish electrochemical kinetics.

The long-term cycling stability of the optimized CoP-400 electrode was systematically investigated to evaluate its structural durability and electrochemical reliability under continuous high-rate operation. The cycling performance was examined through repeated GCD measurements conducted at a high current density of 100 mA/cm^2^ over 12,000 consecutive cycles, and the corresponding evolution of capacitance retention and coulombic efficiency is presented in Figure 9d. During the initial stage of cycling, the electrode exhibits a slight increase in capacitance, which is commonly associated with an electrochemical activation process. This phenomenon may be associated with electrochemical activation, progressive wetting of the electrode surface, gradual electrolyte accessibility to inner porous regions, and activation of electroactive cobalt sites, thereby enhancing the overall utilization efficiency of the active material [41]. Following the activation region, the CoP-400 electrode displays highly stable electrochemical behavior with only negligible capacitance decay throughout the prolonged cycling process, indicating excellent reversibility of the Faradaic redox reactions. After completion of 12,000 charge–discharge cycles, the electrode retains approximately 84.44% of its initial capacitance, corresponding to a minimal capacitance loss of only 15.56%. The remarkable cycling durability demonstrates the superior structural integrity and chemical stability of the thermally optimized CoP framework under repeated ion insertion/extraction processes. In addition to capacitance retention, the CoP-400 electrode maintains a consistently high coulombic efficiency throughout the cycling test, reaching approximately 91.58% after 12,000 cycles. The stable coulombic efficiency confirms the highly reversible nature of the Faradaic charge-storage mechanism and indicates negligible parasitic side reactions occurring at the electrode/electrolyte interface. The efficient charge recovery observed during repeated cycling further demonstrates the excellent electrochemical reversibility and fast reaction kinetics of the optimized CoP electrode [42]. Overall, the combination of high capacitance retention, stable coulombic efficiency, and excellent structural resilience clearly demonstrates the exceptional long-term electrochemical durability of the CoP-400 electrode. These findings confirm that controlled annealing-temperature engineering plays a critical role in stabilizing the CoP framework and enhancing its resistance against electrochemical degradation, thereby making CoP-400 a highly promising candidate for durable high-performance supercapacitor applications.

## 5. Electrochemical Performance of Asymmetric Supercapacitor Device

**Figure 10:**
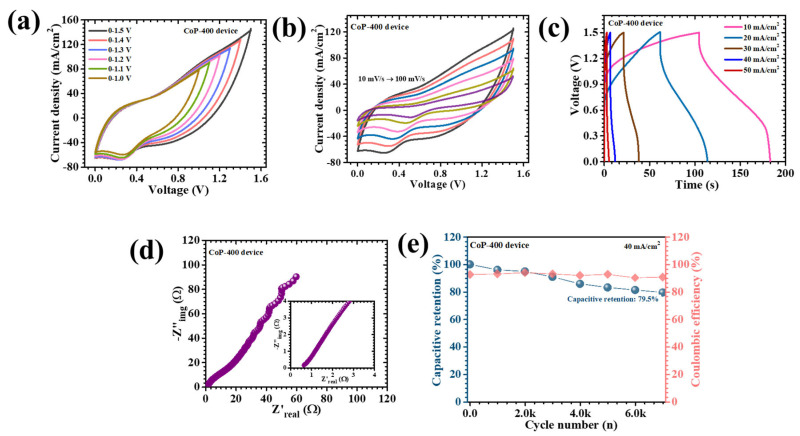
Electrochemical performance of the CoP-400//AC asymmetric supercapacitor: (**a**) CV curves at different voltage windows, (**b**) CV profiles at various scan rates, (**c**) GCD curves, (**d**) Nyquist plot, and (**e**) cycling stability.

To evaluate the practical applicability of the optimized CoP electrode for real energy-storage systems, an asymmetric supercapacitor device (ASD) was successfully assembled using CoP-400 as the positive electrode and activated carbon (AC) as the negative electrode. The combination of Faradaic CoP and electric double-layer capacitive AC was strategically selected to simultaneously achieve high ED and excellent power capability. Both electrodes were fabricated on conductive nickel foam current collectors, while 2 M KOH aqueous electrolyte and porous separator paper were employed to facilitate efficient ion transport and electrical insulation between the electrodes. The electrochemical behavior of the assembled CoP-400//AC asymmetric device was systematically investigated using CV, GCD, and EIS techniques. The operating voltage window of the CoP-400//AC ASD was optimized through CV measurements recorded at progressively expanded potential ranges from 0–1.0 V to 0–1.5 V (Figure 10a). With increasing voltage window, the integrated CV area gradually increased without significant distortion of the curve shape or abrupt current response, indicating excellent electrochemical reversibility and stable device operation. Notably, the device maintained a stable CV profile up to 1.5 V, confirming the absence of severe polarization or electrolyte decomposition within this range. Furthermore, Figure 10b presents the CV curves measured at different scan rates ranging from 10 to 100 mV/s within an extended operating voltage window of 0–1.5 V. The CV profiles exhibit a combination of quasi-rectangular characteristics together with distinct redox humps, indicating the coexistence of electric double-layer capacitance from the AC electrode and diffusion-controlled pseudocapacitive reactions originating from the CoP electrode. Importantly, the device maintains stable electrochemical behavior throughout the wide voltage range without obvious distortion of the CV profiles, confirming excellent reversibility and electrochemical compatibility between the positive and negative electrodes.

The GCD profiles of the CoP-400//AC device, recorded at different current densities, are displayed in Figure 10c. The nonlinear charge–discharge behavior accompanied by slight voltage plateaus further confirms the dominant pseudocapacitive contribution associated with reversible cobalt redox reactions. At a current density of 10 mA/cm^2^, the asymmetric device delivers a high areal capacitance of 302 mF/cm^2^, together with an ED of 0.094 mWh/cm^2^ and an ED of 1.96 mW/cm^2^ (Table 3). The superior electrochemical performance of the device can be attributed to the synergistic integration of the highly redox-active CoP-400 electrode with the highly conductive AC electrode, which collectively facilitate rapid electron transport and efficient electrolyte-ion diffusion during the charge–discharge process. EIS was further conducted to analyze the interfacial resistance and charge-transfer characteristics of the assembled device. As shown in Figure 10d, the Nyquist plot exhibits a relatively small semicircle in the high-frequency region followed by a steep linear response in the low-frequency region, indicating low charge-transfer resistance and efficient ion-diffusion kinetics. The ESR of the CoP-400//AC device was determined to be 0.633 Ω, reflecting excellent electrical conductivity and favorable electrode/electrolyte contact. Moreover, the long-term cycling durability of the asymmetric device was evaluated through continuous charge–discharge cycling at a current density of 40 mA/cm^2^ for 7000 cycles, as illustrated in Figure 10e. The CoP-400//AC device retains approximately 79.5% of its initial capacitance after prolonged cycling while maintaining a high coulombic efficiency of 90.75%. The excellent cycling stability demonstrates the robust structural integrity and electrochemical reversibility of the optimized CoP electrode during repeated redox reactions. The interconnected porous framework effectively accommodates volume fluctuations associated with continuous ion insertion/extraction, thereby minimizing structural degradation and preserving stable electrochemical performance over extended cycling operation. Overall, the outstanding electrochemical characteristics of the CoP-400//AC ASD, including wide operating voltage, high areal capacitance, low internal resistance, excellent rate capability, and remarkable cycling stability, clearly demonstrate the effectiveness of annealing-temperature-engineered CoP electrodes for advanced high-performance energy-storage applications.

## 6. Conclusions

In this study, annealing-temperature-regulated CoP electrodes were successfully developed through hydrothermal growth followed by controlled thermal treatment to understand the influence of structural evolution on charge-storage behavior, ion transport, and electrochemical performance. Among the prepared electrodes, CoP-400 exhibited the most favorable structural features, consisting of interconnected plate-like architectures with balanced crystallinity and open ion-transport channels. Such structural characteristics significantly improved electrolyte accessibility and facilitated rapid charge transfer during electrochemical operation. As a result, the optimized CoP-400 electrode achieved a high areal capacitance of 28.62 F/cm^2^ at 20 mA/cm^2^, together with excellent cycling stability by retaining 84.44% capacitance after 12,000 cycles. Kinetic investigations further confirmed enhanced diffusion-assisted charge storage and reduced internal resistance for the thermally optimized electrode. In addition, the fabricated CoP-400//AC asymmetric supercapacitor demonstrated promising device performance, delivering an areal capacitance of 302 mF/cm^2^, an ED of 0.094 mWh/cm^2^, and a PD of 1.96 mW/cm^2^, while maintaining stable cycling behavior over prolonged operation. The findings demonstrate that controlled annealing provides an effective strategy for tailoring the structural and electrochemical properties of CoP electrodes, offering strong potential for advanced asymmetric supercapacitor applications.

## Figures and Tables

**Table 1:** Summary of kinetic and electrochemical parameters, including diffusion coefficients, b-values, and ESR of the investigated electrodes.

Sample Code	Diffusion Coefficient (cm^2^/s) × 10^−6^		b-Value	ESR (Ω)
	Oxidation	Reduction		
**CoP-300**	2.28	1.88	0.37	0.53
**CoP-400**	7.94	6.98	0.41	0.39
**CoP-500**	4.55	3.94	0.38	0.47

**Table 2:** Electrochemical performance evaluation of the electrodes, presenting areal capacitance, areal capacity, ED, and PD at different current densities.

Sample Code	I (mA/cm^2^)	Areal Capacitance C_A_ (F/cm^2^)	Capacity (mAh/cm^2^)	ED (mWh/cm^2^)	PD (mW/cm^2^)
**CoP-300**	20	10.31	0.642	0.289	4.44
	30	10.27	0.630	0.283	6.62
	40	10.11	0.617	0.278	8.97
	50	10.00	0.617	0.278	10.99
	60	9.87	0.593	0.267	12.97
	70	7.47	0.432	0.194	14.58
**CoP-400**	20	28.62	1.642	0.739	4.13
	30	25.40	1.481	0.667	6.30
	40	23.73	1.358	0.611	8.24
	50	21.89	1.235	0.556	10.15
	60	20.27	1.148	0.517	12.24
	70	19.29	1.080	0.486	14.11
**CoP-500**	20	16.98	1.111	0.500	4.71
	30	15.80	1.019	0.458	6.96
	40	14.67	0.938	0.422	9.21
	50	13.78	0.864	0.389	11.29
	60	13.07	0.815	0.367	13.47
	70	12.13	0.735	0.331	15.26

**Table 3:** Energy storage performance metrics of the CoP-400//AC asymmetric supercapacitor device.

Sample Code	I (mA)	Areal Capacitance C_A_ (mF/cm^2^)	Capacity (mAh/cm^2^)	ED (mWh/cm^2^)	PD (mW/cm^2^)
**CoP-400//AC** **device**	10	302	0.063	0.094	1.96
	20	276	0.057	0.086	3.27
	30	133	0.028	0.042	4.41
	40	36	0.007	0.011	4.00
	50	18	0.004	0.006	5.00

## Data Availability

The data presented in this study are available upon request from the corresponding author.
